# Renal Agenesis, Extramural Ectopic Ureter, and Nonfunctioning Urinary Bladder: A Difficult Clinical Case with an Innovative Approach

**DOI:** 10.1155/2023/3885397

**Published:** 2023-07-04

**Authors:** Armands Vekšins, Laura Voiko, Charlotte Sandersen, Ilze Dūzena, Olga Rabočaja

**Affiliations:** ^1^Latvia University of Life Sciences and Technologies Faculty of Veterinary Medicine, Jelgava, Latvia; ^2^The University of Liege Faculty of Veterinary Medicine, Liege, Belgium

## Abstract

*Summary*. A 7-month-old female Jack Russell Terrier weighing 4.6 kg was referred to a veterinary hospital for persistent urinary incontinence. Blood test results and vital signs were within the normal range. Computed tomography allowed the diagnosis of extramural ectopic ureter and unilateral renal agenesis. After the first neoureterocystostomy surgery, the dog had severe complications, such as hydroureter and hydronephrosis, so a second surgery was performed. A commercial ureteral stent was not an option, and it was decided to fabricate a homemade stent to avoid euthanasia. The stent used was a soft, DEHP-free PVC tube with a lumen of 3 × 4.1 mm and a length of approximately 40 mm that connected the ureter to the urinary bladder. Two years after surgery, the dog is in good general condition, and the results of regularly performed blood and urine tests are within the normal range for dogs.

## 1. Introduction

Congenital diseases of the urinary system can be observed in both the upper and lower urinary tracts [1, 2]. One of the most common congenital disorders of the upper urinary tract is ureteral ectopia [2], but renal dysplasia and agenesis are also relatively common [3–5]. Renal agenesis is the absence of one or both kidneys, and it can occur in different animal species [6–9]. The clinical signs of animals with renal agenesis may vary, and in some cases, the diagnosis may be made incidentally [10]. The situation is different in dogs with ureteral ectopia, as this condition is usually manifested by urinary incontinence [10]. The diagnosis of ureteral ectopia can be made by ultrasonography, computed tomography, and radiography [11, 12]. Surgery is recommended as an effective treatment to prevent incontinence, bacterial infections, and secondary renal changes [13]. Surgical treatment of an ectopic ureter includes neoureterostomy, neocystoureterostomy, combined neocysteureterostomy and neoureterostomy, and ureteronephrectomy, which is recommended for end-stage hydronephrosis [13]. After surgery, most dogs recover completely, but complications may occur in some cases [13]. The most common complications of these surgical procedures are hydroureter and hydronephrosis [14].

Here, we present the surgical procedures and complications encountered in a case of renal agenesis and unilateral ectopic ureter in a 7-month-old Jack Russell Terrier.

## 2. Case Presentation

### 2.1. Anamnesis

A 7-month-old female Jack Russell terrier weighing 4.6 kg was referred to Latvia University of Life Sciences and Technologies Veterinary Hospital (LBTU) with a history of persistent urinary incontinence. Previously performed blood tests were without significant changes, and abdominal ultrasound showed a dilated right ureter and an absent left kidney.

### 2.2. Clinical Examination and Computed Tomography

Vital signs were within normal limits. The perineal area was moist without skin inflammation. A computed tomography scan was then performed using a 16-slice MDCT scanner (Philips MX-16). The dog was premedicated with butorphanol 0.25 mg/kg intravenously (i.v.) and induced with propofol 4 mg/kg i.v. after 15 minutes, and maintained with inhalation anesthesia (isoflurane). During the examination, the dog was positioned in the sternal position, and high-resolution CT scans (120 kV; 259 mA; 2 mm thickness, WL40; WW 350) were performed. Native and postcontrast images (Ultravist, 623 mg/ml (300 mg/ml iodine), 2 ml/kg i.v.) of venous and delayed phases of 2, 4, and 10 minutes were obtained.

**Figure 1:**
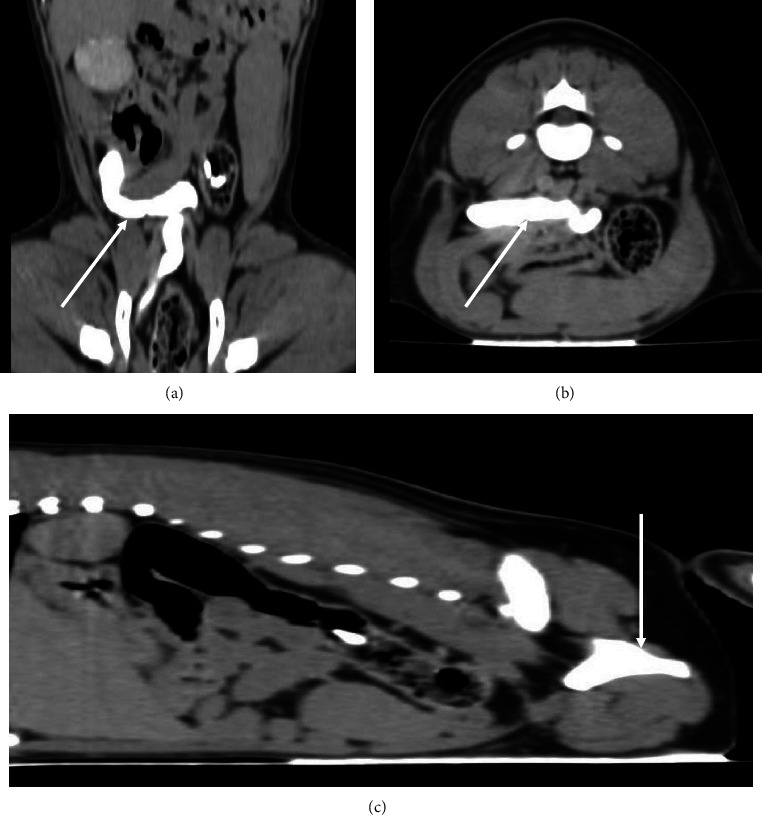
Postcontrast computed tomography scan images using the soft tissue window (WW 350; WL40). Dorsal (a), transverse (b), and sagittal (c) planes. Observe the contrast medium on the right ureter (arrows).

Computed tomography showed an enlarged right kidney (49.1 × 30.4 mm) with changes in shape; however, the renal pelvis (<2 mm) and medullary part were without significant abnormality. The ureter was dilated throughout its length (cranially 14.4 mm; centrally 19.8 mm; and 6.2 mm in the caudal part at the level of the urinary bladder) and opened into the vagina (Figure 1). The urinary bladder was reduced in size (30.2 × 14.8 mm) without intramural contents, and the left kidney was absent. Conclusion from CT included unilateral renomegaly, hydroureter, ectopic ureter, and agenesis of the left kidney.

According to CT, surgical treatment was recommended, and the owner was informed of the doubtful prognosis and associated risks. Surgical treatment was recommended to avoid urinary incontinence and possible future risks such as bacterial infections and secondary renal damage. The owner agreed to surgical treatment, and the day of surgery was set.

### 2.3. Surgery and Postoperative Treatment

#### 2.3.1. Anesthesia

On the day of surgery, the dog's vital signs were within normal limits, and blood test results showed no significant changes. A 5-minute preoxygenation with 100% oxygen was performed, and the patient was anesthetized according to the standard PIVA protocol. The anesthetic protocol included premedication with acepromazine 0.01 mg/kg i.v. and fentanyl at 3 mcg/kg i.v., followed by a CRI of 5-10 mcg/kg/h. Midazolam 0.2 mg/kg i.v. was used as a coinduction agent, followed by induction with propofol 4 mg/kg. After orotracheal intubation, general anesthesia was maintained with isoflurane in 100% oxygen. The positive inotropic drug dopamine 5-10 mcg/kg/min i.v. was administered to achieve mean arterial pressure (MAP) in the normal range. An Intravenous infusion of Ringer's lactate solution 3 ml/kg/h was administered throughout the surgical procedure. A urinary catheter was placed before surgery to assess urinary output (UO). Systemic antibiotics amoxicillin with clavulanic acid 20 mg/kg i.v. were administered. An electrocardiogram on lead II was used to continuously monitor heart rate (HR), pulse oximetry, end-tidal carbon dioxide, noninvasive MAP, and esophageal temperature with the BM3Vet multiparameter veterinary monitor.

#### 2.3.2. Surgery I

A celiotomy was performed through a caudal median incision. Initially, both ovaries and uterine horns were found, and an ovariohysterectomy was performed. Inspection of the abdomen revealed a missing left kidney and a dilated right ureter that paralleled the urinary bladder and ended in the pelvic cavity. To connect the ureter to the urinary bladder, neoureterocystostomy was chosen as the surgical method. Second, a cystotomy was performed, and the right ureter was ligated and transected near the pelvic cavity. An approximately 8 mm opening was created in the right dorsal wall of the urinary bladder, and the distal ureter was inserted into the blade and sutured to the bladder mucosa in a simple, interrupted fashion using polydioxanone monofilament absorbable suture material. The cystotomy incision was closed in two layers with a continuous pattern. The urine flow through the catheter was normal.

#### 2.3.3. Postoperative Care I

**Figure 2:**
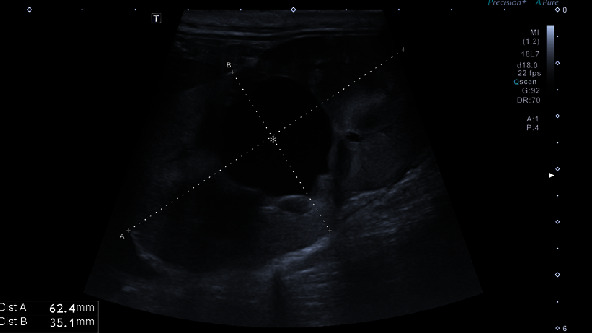
Ultrasound image of the right kidney. Dilated pelvis (asterisk).

**Figure 3:**
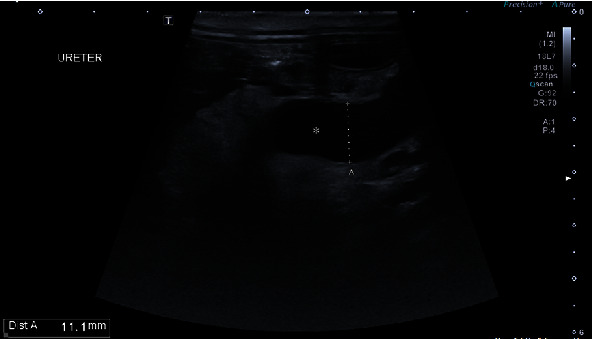
Ultrasound image of the proximal part of the right ureter (asterisk).

**Figure 4:**
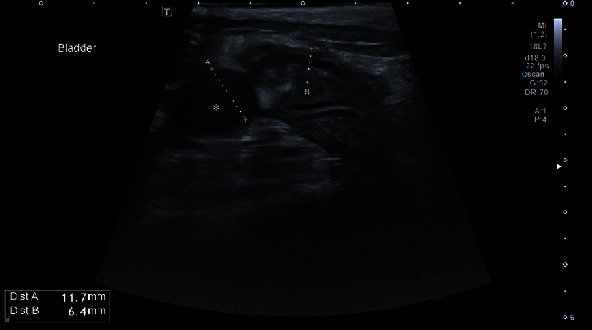
Ultrasound image of the right proximal ureter (asterisk) at the level of the urinary bladder.

After surgery, maropitant 1 mg/kg i.v., famotidine 1 mg/kg i.v. q12, amoxicillin+clavulanic acid 20 mg/kg q12, buprenorphine 0.02 mg/kg i.v. q6h, meloxicam 0.2 mg/kg and Ringer lactate CRI 3 ml/kg/h were administered. On the day of surgery, a small amount of red urine was passed through the urinary catheter. On the first day after surgery, the animal was responsive but slept most of the day and refused to eat. The meloxicam dose was reduced to 0.1 mg/kg, but other medications were used according to the treatment plan. Blood tests revealed elevated levels of urea (18.02 mmol/l), creatinine (148.0 mmol/l), and leukocytosis (WBC 26.1 103/*μ*l). A small amount of red urine was excreted through the urinary catheter. The next day, the animal was depressed, no urine was excreted, and blood tests showed elevated urea (28.98 mmol/l), creatinine (251.64 mmol/l), phosphorus (2.61 mmol/l), sodium (156.9 mmol/l), WBC (23.g103/*μ*l), RBC (5.1 103/*μ*l), and PCV 36.8%. The patient was sent to the ultrasound department, where severe hydronephrosis was found in the right kidney (Figure 2). The renal pelvis and diverticula of the kidney were distended, rounded, and filled with anechoic fluid. Distally, acoustic enhancement was visible. The renal pelvis measured up to 21.9 mm. The kidney was hyperechoic, and the corticomedullary junction was indistinct. The dimensions of the kidney were 62.4 × 35.1 mm. The longitudinal section was taken at the proximal part of the right ureter (Figure 3). The right ureter was dilated and filled with anechoic fluid. The diameter of the proximal ureter was 11.1 mm. It was easy to follow it caudally to the level of the urinary bladder wall (Figure 4). Distended distal ureter adjacent to the urinary bladder. The lumen of the distal ureter measured up to 11.7 mm. The urinary bladder was hypoplastic. The wall was thickened (6.4 mm), and no urine was seen in its lumen. Based on the ultrasonographic findings, repeat surgery was recommended to reevaluate the surgical site and treat the narrowing of the ureter.

#### 2.3.4. Surgery II

**Figure 5:**
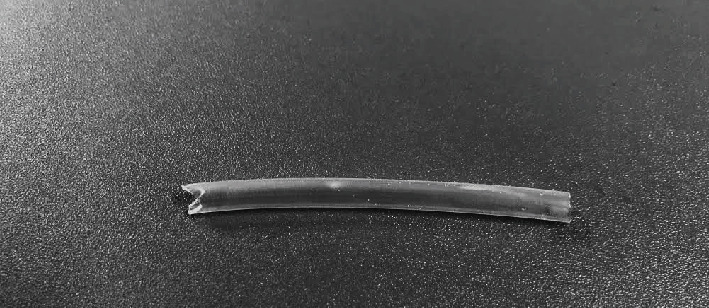
A soft, DEHP-free PVC tubing stent similar to the one used during surgery.

Laparotomy was performed through the same incision. Compared with the first operation, the ureter was more dilated, especially the distal part connected to the urinary bladder, and a small amount of urine leakage through the stoma was noted. After cystotomy, urine flow was observed, and it was thought that the cause of obstruction was the size of the urinary bladder and the thick mucosa that obstructed urine flow. Initially, part of the mucosa was removed at the site of the stoma, and the serosa of the ureter was sutured to the serosa of the urinary bladder from the outside to prevent urine leakage on the side of the stoma. The cystotomy was closed as usual, and before abdominal closure, the ureter was rechecked, and it was determined that the urinary flow was insufficient to prevent further dilatation. Because the standard procedure was ineffective, other solutions were discussed. A commercial ureteral stent was not an option, and it was decided to fabricate a homemade stent to avoid euthanasia. The stent used was a soft, DEHP-free PVC tube with a lumen of 3 mm and a length of approximately 40 mm. The size of the stent was chosen so that part of it would fit into the ureter and part into the bladder. It was important that it was not too long, and that the bladder mucosa did not occlude it. A stent like the one used during surgery is shown in Figure 5. Part of the stent was placed in the urinary bladder and the other part in the ureter. On both sides, the stent was attached to the inner wall of the urinary bladder with a simple, interrupted suture. The urinary bladder was closed as usual, and the ureter was checked before closing the abdominal wall. The ureter was soft, and no further distension was noted. The laparotomy incision was closed as previously described.

#### 2.3.5. Postoperative Care II

After surgery, maropitant 1 mg/kg i.v., famotidine 1 mg/kg i.v. q12, amoxicillin+clavulanic acid 20 mg/kg q12, fentanyl 0.2 mg/kg/h CRI, and Ringer lactate CRI 2 ml/kg/h were administered. After surgery, the dog was responsive, and vital signs were within normal range. Urine flow was observed and measured with a urine drainage bag. The average urine output was approximately 400 ml per day. The urinary catheter was removed on the fifth day. In the postoperative period, blood tests were repeated on days 2, 4, and 6, and urea, creatinine, sodium, potassium, phosphorus, and leukocytes were determined. On discharge day, urea, creatinine, and potassium were redetermined. The results of the laboratory tests are shown in Table 1. In the postoperative period, urinary ultrasonography was repeated daily, and the findings were without significant changes compared with the examination before the second surgery; however, the urinary bladder was filled with fluid. On the day of discharge, the dog was responsive with normal vital signs, good appetite, and laboratory values in the normal range.

#### 2.3.6. Recheck Visits

At the time of discharge, amoxicillin+clavulanic acid 20 mg/kg q12 were given orally for two days, VetExpert RenalVet supplement for one month, surgical wound care, and repeat blood tests after 2 days, and ultrasound after one month were recommended.

After two days, blood test results were within normal limits (urea 9.48 mmol/l, creatinine 71.19 mmol/l, and leukocytes 11.1 103/*μ*l), the dog was responsive, had a good appetite, and had normal urination. Other recommendations were ultrasound and blood tests after months.

One month later, ultrasonography did not reveal any significant changes compared with the previous examination. The kidney measured 51 by 29 mm. The renal pelvis was slightly enlarged, from 9 mm to 13 mm, and the proximal ureter measured 12 mm. A small amount of hyperechoic sediment was found in the ureter near the urinary bladder. Blood test results were within normal limits with no significant changes from the last visit.

**Figure 6:**
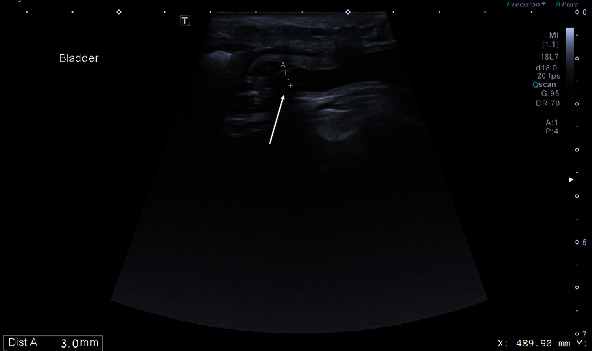
Ultrasound image of the right proximal ureter and stent (arrow).

After three months, ultrasound and blood tests were performed. Ultrasound results showed that the kidney was hyperechoic, the corticomedullary junction was indistinct, and hydronephrosis was still present, but the dimensions of the renal pelvis decreased to 12.1 mm. The size of the kidney decreased to 50.7 × 28.6 mm. The proximal ureter measured 10.9 mm. There was no significant decrease in size compared with the previous ultrasound. The wall thickness of the urinary bladder decreased sharply, reaching 1.2 mm (decrease of 5.2 mm). It was filled with anechoic fluid, corresponding to the normal echogenicity of urine. Because normal bladder wall thickness varies with the degree of distension, the thinning of the urinary bladder wall in this case could be explained by improved bladder function. Two parallel hyperechoic lines were visible in the urinary bladder. These lines corresponded to the stent that was inserted during surgery. The diameter of the stent was 3 mm (Figure 6). At the time of ultrasonography, cystocentesis was performed and urine samples were obtained, the results of which were within normal limits. Blood test results showed a slightly elevated urea level (14.48 mmol/l); however, the creatinine level was normal (61.33 mmol/l). After the second follow-up visit, it was recommended that the blood and urine tests be repeated every three months at the local veterinarian and transmitted to the LBTU Veterinary Hospital if necessary.

### 2.4. Outcome

Two years after the operation, the dog is alive and responsive, and the results of blood and urine tests performed regularly are in the normal range.

## 3. Discussion

We presented a difficult case of extramural ectopic ureter and renal agenesis in a dog. Although ureteral ectopia is one of the most common congenital urinary disorders in dogs [2], the treatment in this case was complicated and required an innovative approach. In our case, the owner complained of urinary incontinence of his dog, and this is consistent with previously published information [10]. Since the dog had unilateral renal agenesis and the other kidney had an ectopic ureter, a complete analysis was performed to decide on surgical treatment. In cases like this, it is not only the choice of the right surgical technique that matters but also the choice of the anesthetic protocol. The choice of opioid should be based on the anticipated level of pain for the procedure to be performed. Full *μ* opioid receptor agonists such as methadone or morphine or fentanyl are appropriate for procedures that are expected to cause moderate to severe pain [15]. In healthy animals, especially after intravenous injection, midazolam or diazepam can cause disinhibition and excitation, but they often sedate sick animals. In uremic patients, lower doses should be administered, titrated according to effect. Because the effects and half-life of drugs in humans with renal dysfunction are unknown, drugs that are primarily excreted unchanged by the kidneys or that have active metabolites that must be excreted by the kidneys should be avoided [16]. It is possible to maintain anesthesia with volatile anesthetics such as isoflurane, sevoflurane, and desflurane [17], although they all cause dose-dependent vasodilation and hypotension. To administer lower concentrations of volatile agents, balanced anesthetic techniques should be used, such as intravenous infusion of fentanyl.

Several surgical techniques are described, such as neoureterostomy, ureteroneocystostomy, and neocystoureterostomy [13, 18]. Our decision to perform end-to-side neoureterocystostomy was based on the surgeon's experience and the opinion of other authors that neoureterocystostomy is a good choice for the treatment of extramural ectopic ureters [19]. After the first operation, the dog had complications that could be classified as major. Complications are not rare, but in 72% of cases they are minor, e.g., hematuria, dysuria, and stranguria, and only in 8% of cases the animals have major complications, such as uroabdomen, ureteral obstruction, or stenosis [13]. In the postoperative period, it is important to hospitalize and monitor patients. If serious complications are diagnosed, immediate action must be taken. In our case, the goal of the second operation was to stop hydronephrosis of the right kidney and ensure urine flow. During the operation, it became clear that the cause of hydronephrosis and azotemia was ureteral obstruction caused by the bladder mucosa. Stenting of the ureter may be an option for the initial treatment of dogs with benign ureteral obstruction [20]. In veterinary practice, polymeric materials are the main starting material for ureteral stents. Postoperative complications can occur with stents, such as restenosis, stent migration, or tissue encrustation. To reduce complications, work is being done to improve biocompatibility, durability, and flexibility [21]. Normally, commercial stenting material should be used, but in our case, it was not available, and since an urgent solution was needed, it was decided to use a soft DEHP-free PVC tube as a stent. Ureterocolonic anastomosis was not considered because of the poor results and high morbidity [22]. The idea was to implant the stent for a short period of time until the urinary bladder started to function properly (inflate, hold, and release urine). After the second postoperative period, the dog was carefully monitored by checking renal waste products and inflammation. Dogs should be monitored for urinary tract infections after stenting [20]. Because our stent material was homemade, we raise possible complications such as side effects of the PVC tube, infection, stenosis, and stent displacement.

Two years later, the dog is alive with no clinical signs of kidney disease, the stent is still in the implanted site, and blood and urine tests are in the normal range.

## 4. Conclusion

The management of an ectopic ureter can be challenging in some cases, and when complications arise, a rapid response is required. We do not recommend the technique we describe as a standard procedure; however, sometimes an innovative approach is needed to save lives.

## Figures and Tables

**Table 1:** Blood test results.

Day	Urea (mmol/l) (WRR 3.6-10.00)	Creatinine (mmol/l) (WRR 44-133)	Sodium (mmol/l) (WRR 141.1-152.3)	Potassium (mmol/l) (WRR 3.9-5.65)	Phosphorus (mmol/l) (WRR 0.5-2.6)	WBC (10^3^/*μ*l) (WRR 6.0-17.0)
Two days after surgery	33.01^∗^	211.83^∗^	152.2	4.44	2.57^∗^	23.6^∗^
Four days after surgery	11.76^∗^	102.43	150.0	4.25	2.2	20.4^∗^
Six days after surgery	12.32^∗^	91.31	146	3.8^D^	2.1	11.9
Seventh day after surgery	7.67	81.15	—	4.53	—	—

^∗^Elevated; ^D^Deficiency; WRR: values within the reference range.

## Data Availability

The data used to support the findings of this study are included in this article.
